# Just a Reflection: Does Drug Repurposing Perpetuate Sex-Gender Bias in the Safety Profile?

**DOI:** 10.3390/ph14080730

**Published:** 2021-07-27

**Authors:** Ilaria Campesi, Giorgio Racagni, Flavia Franconi

**Affiliations:** 1 Department of Biomedical Science, University of Sassari, 07100 Sassari, Italy; 2 National Laboratory of Pharmacology and Gender Medicine, National Institute of Biostructure and Biosystem, 07100 Sassari, Italy; franconi.flavia@gmail.com; 3 Department of Pharmacological and Biomolecular Sciences, University of Milan, 20133 Milan, Italy; giorgio.racagni@unimi.it

**Keywords:** sex, gender, inflammation, pharmacokinetics, COVID-19

## Abstract

Vaccines constitute a strategy to reduce the burden of COVID-19, but the treatment of COVID-19 is still a challenge. The lack of approved drugs for severe COVID-19 makes repurposing or repositioning of approved drugs a relevant approach because it occurs at lower costs and in a shorter time. Most preclinical and clinical tests, including safety and pharmacokinetic profiles, were already performed. However, infective and inflammatory diseases such as COVID-19 are linked with hypoalbuminemia and downregulation of both phase I and phase II drug-metabolizing enzymes and transporters, which can occur in modifications of pharmacokinetics and consequentially of safety profiles. This appears to occur in a sex- and gender-specific way because of the sex and gender differences present in the immune system and inflammation, which, in turn, reflect on pharmacokinetic parameters. Therefore, to make better decisions about drug dosage regimens and to increases the safety profile in patients suffering from infective and inflammatory diseases such as COVID-19, it is urgently needed to study repurposing or repositioning drugs in men and in women paying attention to pharmacokinetics, especially for those drugs that are previously scarcely evaluated in women.

## 1. Introduction

Repurposing or repositioning old medications to treat new diseases or unmet needs is an attractive and alternative form of drug discovery [1]. The oldest and very successful repurposed drug is acetylsalicylic acid. Initially, in 1899, it was marked as an analgesic, but, in 1980, it was repositioned as an anti-aggregating agent, at low doses, thanks to the research of Sir John Vane [2,3]. For this research, in 1982, he became a Nobel laureate in medicine. Perhaps in the very near future the aspirin could be again repurposed in oncology. Daily treatment with acetylsalicylic acid for some years can prevent the development of many cancers including colon-rectal cancer [4]. The non-selective adrenergic beta-blocker propranolol, which has been used for decades as a cardiovascular drug since 2004, is the first-choice therapy for infantile hemangioma, and it is the only approved drug for complicated hemangioma [5]. Finally, thalidomide was repurposed twice. It was launched in 1957 as a sedative or tranquilizer but was soon used throughout the world, except in the US, for treating morning sickness in pregnant women. However, in 1962, it was banned from the World Health Organization for its teratogenicity because it was calculated that over 10,000 babies born by mothers treated with thalidomide present phocomelia [6]. The thalidomide tragedy prompted a revision of pre-marketing toxicity tests. However, some years later its efficacy against *erythema nodosum leprosum*, a complication of leprosy, was evidenced, and, in 1998, it was repurposed as an orphan drug for complications of leprosy [7]. Because of thalidomide antiangiogenic properties, in 2006, its second repositioning occurs as a first-line agent for multiple myeloma, and because of its known teratogen effects, the thalidomide dispensing is regulated by the System for Thalidomide Education and Prescribing Safety program [8].

**Figure 1:**
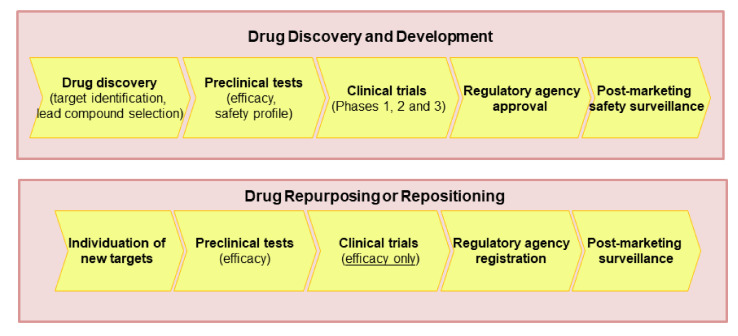
The main differences between drug discovery and development and drug repurposing processes.

Nevertheless, the reported examples, the concept of repurposing (which is also called repositioning, reprofiling, redirecting, switching, etc.) emerged in 2004 [9], when drug repositioning was defined as the ability to find new indications for an old drug. Later, the definition was expanded and now includes drugs withdrawn from the market for safety issues, active molecules that failed the clinical phases for toxicity, or for low efficacy, excluding molecules that have not been clinically tested [10]. The repurposing or repositioning of medications can help in individuating new treatments for diseases at a lower cost and in a shorter time (Figure 1) because most preclinical and clinical tests, including safety and pharmacokinetic profiles, were already performed [11,12,13]. Further, some countries such as the US have a simplified procedure for the introduction on the market of repositioned drugs [10].

Three main steps characterize repurposing: recognition of the drug, efficacy of the drug in preclinical and clinical investigation [14].

Some concerns affect the selection of the optimal dose because the previous drug knowledge may not be suitable for the new indication [10,11,12,14,15]. This is relevant because the level of tolerable safety depends on its indications. For example, the adverse drug effects could be less acceptable if the repositioned agent will be used to treat a less severe disease than the original one [16]. In addition, the common use of the paradigm “one dose fits all”, neglecting the sex differences in body dimension and composition, metabolism, and elimination [17], may lead to the risk of women’s overmedication contributing, at least in part, to women-biased adverse drug reactions (ADR) [18].

A challenge concerns intellectual property. The repurposed drugs have relatively weak intellectual protection, and this is related only to the indication and patent on indications [19]. They are also a potential legal challenge on the basis that the new indication was predictable from data in the scientific literature [16].

The recent COVID-19 pandemic induced by coronavirus SARS-CoV-2 has raised serious global concerns for public health and constitutes a societal and economic emergency all over the world. Unfortunately, no specific drugs are available to fight this pandemic, leading to thousands of deaths over the world. There is a large interest to accelerate the discovery and/or development of active and safe drugs towards this pandemic. As already mentioned, the value of drug repurposing is to speed up the traditional process of drug discovery by identifying a novel clinical use for drugs that have already proven to be safe and effective in humans and are approved for other indications.

The efficacy and safety profiles of several drugs are deeply affected by sex and gender [17,18,20,21,22]. These are two specific but inseparable concepts that often interact in a continuous, multi-dimensional, entangled manner. Sex is limited to the biological body [23,24,25,26]. Genes and sexual hormones have pivotal roles in determining male and female phenotypes [20,27,28,29]. Gender definition is more difficult, and it includes socioeconomic status, income, education, neighborhood characteristics, lifestyles such as smoking, environmental exposures including drugs, access to healthcare, and other social determinants of health [30]. The social differences between women and men depend on the single society and culture and are changeable over time, being different among countries and cultures. In other words, beyond the genes and hormones, the experience of life such as smoking may modify male and female phenotypes [31,32]. Some authors have proposed the term sex- and gender-based medicine [33,34], whereas others have proposed sex- and gender-specific health [33]. In this study, in accordance with other authors [33,34,35,36], we use the term “sex and gender”, which recognizes the value of both the biological and social contest as already stated by other authors [17,37].

Men and women have both sex-specific inflammation and sex-specific immunity [15,38,39] (see also below), even if the biological basis of this sexual dimorphism is not yet fully clear. In addition, COVID-19 (see also below) appears sexually dimorphic [40,41,42,43].

This review aimed to verify whether the repurposed drugs proposed for COVID-19 focus on the sex and gender differences observed in the pandemic and drug response. In other words, this review aimed to verify if the drug data obtained in COVID-19-free subjects can be translated to COVID-19 patients, especially in critically ill individuals. In addition, it is stressed that it is mandatory to consider sex and gender as a variable for ameliorating the clinical management of COVID-19 patients and to increase the safety profile.

## 2. Drugs Candidates for Repurposing in COVID-19 Infection

Numerous medications approved for other diseases have been tested and/or are still tested. Overall, they involve (a) specific drugs (agents under investigation or reported to have effects against COVID-19 inhibiting one or more steps of the coronavirus lifecycle; (b) medications that may help cure the effects of COVID-19 patients such as infection and massive inflammation, which lead to severe complications such as coagulopathy and acute respiratory distress syndrome [44]. Currently, the FDA has approved three therapies: (a) convalescent plasma, anti-Ebola agent: remdesivir alone or in association with baricitinib, and two monoclonal antibodies: casirivimab/imdevimab (REGN-COV2) [45]. However, there are some concerns related to their efficacy [46].

### 2.1. Drugs Inhibiting One or More Steps of SARS-CoV-2 Lifecycle: Virus Attachment and Entry

The virus enters the cells via endocytosis or through the interaction with the spike (S) protein of the virus and angiotensin-converting enzyme 2 (ACE2) and transmembrane protease serine 2 (TMPRSS2) [47]. It was also hypothesized that dipeptidyl peptidase 4 (DPP4) could be a functional receptor for the S protein of SARS-CoV-2 [48]; if so, the DPP4 inhibitors may play a role in preventing and decreasing the risk and progression of COVID-19 [49]. Moreover, it was suggested that numerous compounds (estradiol, spironolactone, isotretinoin, and retinoic acid) may down-regulate ACE2 receptors [50], and inhibitors of TMPRSS2 (bicalutamide, camostat mesylate, and nafamostat), inhibitors of DPP4, and inhibitors of endocytotic transport (chloroquine, hydroxychloroquine, amodiaquine, artemisinin and artesunate baricitinib, chlorpromazine, niclosamide, imatinib, and amiodarone) could be useful to treat COVID-19 [50].

### 2.2. Drugs Inhibiting One or More Steps of SARS-CoV-2 Lifecycle: Viral Replication

The replication of the virus requires RNA-dependent RNA polymerase. Some antivirals such as favipiravir, galidesivir, tenofovir, sofosbuvir, and clevudine could inhibit it, whereas other antivirals (remdesivir, emtricitabine) inhibit the replication of RNA [50]. Additionally, some protease inhibitors (atazanavir, danoprevir, darunavir, lopinavir, and ritonavir) used for the treatment of human immunodeficiency virus and acquired immune deficiency syndrome (HIV/AIDS) and the immunosuppressant levamisole have been repurposed for COVID-19 [50]. Other drugs (alfa interferon, beta interferon, and peginterferon lambda, tetracycline derivatives) could inhibit viral reproduction [50].

### 2.3. Drugs Inhibiting One or More Steps of SARS-CoV-2 Lifecycle: Virion Assembly and Release

After the formation, the new virus reaches cell membranes and is released by exocytosis. Some antivirals such as oseltamivir and daclatasvir and the immunosuppressant sirolimus could act at this level blocking the viral replication, and thus they have been suggested for COVID-19 treatment [51,52].

### 2.4. Drugs Potentially Counteracting the Effects of SARS-CoV-2 Infection

COVID-19 is linked with an immune and inflammatory response leading in the severe form of the disease to cytokine storm [53]. This is, in turn, linked with complications like acute respiratory distress syndrome, macrophage activation syndrome, lymphopenia, and coagulopathy [54]. This leads to test anti-inflammatory drugs such as non-steroidal anti-inflammatory drugs, glucocorticoids, kinase inhibitors, and interleukin antagonists [46]. Some macrolide antibiotics are evaluated to be repositioned in COVID-19 [46]. To attenuate the respiratory complications, several drugs (nintedanib, pirfenidone, pamrevlumab, bevacizumab, aviptadil, eculizumab, and conestat alfa) are currently being evaluated [46]. Fibrinolytic therapy has been proposed to threat the activation of coagulation, and tissue-plasminogen activator and alteplase are under investigation [46]. In addition, some general anesthetics (ketamine, sevoflurane, and isoflurane) have also been proposed to reduce systemic inflammation and acute respiratory distress syndrome severity; the antidepressants (fluoxetine and fluvoxamine) have been proposed to counteract hyper-inflammatory symptoms [46]. Based on the androgen effect on TMPRSS2 expression [55], numerous clinical trials are testing the ability of androgen deprivation therapies or anti-androgens to mitigate COVID-19. Selective estrogen receptor modulators (SERM) are repurposed as anti-viral drugs against the Ebola virus, human immunodeficiency virus (HIV), and HCV infections [56]. Moreover, estrogen receptors are localized in the respiratory tract, and their presence suggests that estrogen may have a role in respiratory viral infections [57]; therefore, SERM could be used for COVID-19.

Vitamin D exerts pleiotropic effects, and its deficiency leads to increased susceptibility to several diseases [58]. Recently, this drug has been repurposed for COVID-19 because low vitamin D status is associated with various degrees of disease severity and mortality [58]. In addition, an observational study shows that mortality is inversely associated with vitamin D supplementation [59]. Interestingly, it interacts with ACE2 (the entry door of virus), attenuates cytokine release, and preserves cell junctions, strengthening cellular immunity [58]. Notably, vitamin D is more active in women with autoimmune diseases than men [60]. The effect of sex on vitamin D levels is unclear, but the majority of data sustains that it is lower in women than in men [61,62,63,64]. Thus, it is plausible that the vitamin D activities could be influenced by sex.

This brief excursus makes clear that a myriad of drugs already used with other indications has been repurposed for this dramatic pandemic.

## 3. Sex and Gender Aspects in COVID-19

In viral infections, sex- and gender-based differences appear to be common. Numerous investigations and governmental data evidenced numerous and relevant sex and gender differences in COVID-19, even if sex-stratified data were reported in only 74 out of 187 countries on the Global Health 5050 [40]. Sex and gender strongly influence the severity and mortality of the disease, which hare higher in men than in women across the lifespan [41,42,43]. COVID-19 is more devastating in old men [53], who have a higher risk for intensive care unit admissions and mechanical ventilation [65]. Further, men die twice as much compared with women [65]. The higher rates of mortality or severity in men are still present after adjusting for comorbidities.

The precise origin of these sex and gender differences is not clear; however, they have been recently summarized [66]. In part, they can depend on biological factors (sex) such as genes, hormones, and diversity in inflammatory host responses (16). Sex differences in immunological and inflammatory diseases persist across all lifespans [38], with the aging of the immune system more pronounced in men than in women [39]. Besides, these differences can be gender-dependent because they involve the social role, lifestyles such as smoking, which is a risk factor, identity, and relations, which play a role, for example, in exposure [65]. Men tend to have habits that could be conducive to viral transmission [67]. Sex and gender differences in access to healthcare facilities may lead to further variability in disease progression [68]. Notably and relevantly, women seem to have more often long-term COVID-19, which persists for longer than 12 weeks, and experience more negative social and economic impacts [69,70]. Understanding the basis of such differences is of great relevance for clinical management [71].

## 4. Sex and Gender Aspects in Drug Response in COVID-19

Generally, women are less enrolled in clinical trials, although with some exceptions [17]; in COVID-19 trials, women only represented ~1/3 of subjects [65]. The under-representation of women has been observed both in randomized clinical trials and in some observational studies [65]. The under-enrollment of women for some authors depends on the lower severity of COVID-19 in women [72]. For others, it depends on ethical reasons linked to fear of teratogen effects. Indeed, in 1977, the Food and Drug Administration (FDA) guidelines excluded women from all trials [73]. Currently, this has completely changed [20,74,75,76].

The low enrollment of women could stem from the assumption that the male perspective represents the norm [37]. This situation leads to poor scientific rigor, and it makes it difficult to compare the efficacy of different therapies [17,20]. Currently, only very few registered clinical trials for COVID-19 in ClinicalTrials.gov present sex and gender as explicit criteria of enrollment or analytical variables [77]. In particular, women are under-enrolled, the outcomes are scarcely disaggregated by sex, and sex and gender differences are inadequately considered in the analysis of the data [78].

Although this is slowly changing, especially in phase 3 trials [79], early-stage trials are still heavily male-biased [20]. Relevantly, there are some studies where the women enrolled prevail over men, especially in phase 3 [80,81]. Therefore, the information deriving from the early stage is mainly missing in women, with some exceptions [81]. This lack of knowledge reflects repurposed drugs.

Growing evidence suggests that men and women may have different pharmacokinetic and pharmacodynamic responses to pharmacological agents [17,21,82,83]. For example, women have a stronger response to vaccinations and more adverse effects than men [82,83].

## 5. Can Male and Female COVID-19 Patients Have the Same Pharmacokinetics as COVID-19 Free Patients?

Beyond physiological differences, chemical and sociocultural aspects affect pharmacokinetic and pharmacodynamic processes [17,20,21]. For example, looking at new drug applications that reported sex analysis: 6–7% reports evidenced sex and gender pharmacokinetic differences over 40% [17]. Currently, pharmacokinetic studies were often performed in healthy subjects, and women were scarcely represented [21] even if numerous sex differences in pharmacokinetics are described [17].

Sex and gender pharmacokinetic differences involve differences in absorption, distribution, drug-metabolizing enzymes of both phase I and II, and transporters, and some of them seem to be sensible to exogenous and endogenous sexual hormones [17,21,22]. For example, in human jejunal and ileal tissues, P-glycoprotein is higher in men than women, influencing the bioavailability of cyclosporine A, a P-glycoprotein substrate [84]. Finally, glomerular filtration, tubular secretion, and tubular reabsorption show sex differences leading to generally higher renal clearance in men than in women [17,21,22].

Bioequivalence studies, which are mainly performed in men, evidence that men and women may have a different response to excipients [22,85]. For example, PEG400 ranging from 0.5 to 1.5 g increases and decreases the bioavailability of ranitidine in men and women, respectively [22].

This sexual dimorphism in pharmacokinetics also occurs in antiviral drugs, and some differences are exemplified in Table 1.

Inflammation can affect pharmacokinetics and pharmacodynamics contributing to variability in drug response. Several investigations illustrated pharmacokinetic alterations in patients with inflammation and infectious diseases [95]. Indeed, inflammation and infectious diseases can alter body fluid distribution, blood protein concentrations, absorption, distribution, metabolism, and excretion of drugs [96,97]. It has long been known that the half-life of theophylline is increased in acute virus infections in asthmatic children [98,99]. During influenza B, several asthmatic children were been hospitalized for ADR induced by treatment with theophylline [100]. Notably, interferon-α in hepatitis B decreases theophylline clearance, increasing its half-life [101]. Interferon therapy is associated with decreased cytochrome P450 (CYP) 1A2 activity, whereas the effect on other CYP enzymes is more variable [95]. Sarilumab and tocilizumab, antibodies against IL-6 receptors, elevate the metabolism of simvastatin and reduce its area under the curve (AUC) [102,103]. 

Globally, acute or chronic inflammation (Table 2) down-regulate both liver and intestinal CYP, carboxylesterases (CES), phase II enzymes, and transporters [95,104,105,106,107] occurring to impaired absorption and pre-systemic and hepatic metabolic biotransformation. IL-6, a good biomarker to test severe cases of COVID-19 [71], plays a pivotal role in the downregulation [108] of CYP (especially CYP1A, CYP3A, CYP2C9, and CYP2C19) and CES1 (Table 2). The inhibition of CES1 reduces the transformation of prodrug oseltamivir, which is more efficiently produced by women’s livers than by men’s [109].

Therefore, the acute or chronic inflammation shifts towards a poorer metabolizing phenotype. This phenotype may be reverted to its physiological status using inhibitors of the inflammatory pathway such as IL-6 monoclonal antibodies [108]. The downregulation of CYP induced by tocilizumab may be present after the tocilizumab suspension [110]. In our opinion, clinicians should be aware that the use of tocilizumab and perhaps the use of other anti-inflammatory drugs might change the activity of CYP enzyme, modifying the pharmacokinetics of drugs, which in turn may occur in inspected drug interactions and food-drug interactions.

In this contest, it is not surprising that COVID-19 patients display much higher concentrations of lopinavir (using ritonavir-boosted lopinavir) than HIV patients treated with the same dose [111,112]. In fact, the estimated dose in COVID-19 patients compared with HIV patients to reach EC50 is about 60- to 120-fold higher [111]. Lopinavir is metabolized by CYP3A4, an enzyme that is more active (20–30%) in females than in males [113]. Therefore, it is also plausible that repurposed medications metabolized by CYP3A4 may present sexual dimorphism in pharmacokinetic, which could also be due to the major inflammation observed in men with COVID-19 than in women [53]. In line with these observations, in animals, inflammation effects on CYP expression appear to be sex and CYP enzyme-dependent [81], while, to the best of our knowledge, we still do not know if sex and gender control this in humans.

Acute and chronic inflammatory responses can induce hypoalbuminemia [114]. In COVID-19, hypoalbuminemia is linked with viral load, severity of acute lung injury, and organ dysfunction [115] and is associated with worse outcomes [116]. Hypoalbuminemia widely influences distribution volume and the therapeutic and safety profiles of medications as only the unbound fraction of the drug is active.

In conclusion, the above data suggest that the inhibition of enzymes and hypoalbuminemia can elevate the exposure to medications that are substrates of inhibited enzymes or reduce the activity of pro-drugs or increase the concentration of free drugs. They also suggest that the pharmacokinetic phenotype is dynamic; in other words, it can be transitory. All this could be strongly influenced by sex and gender.

Importantly, the prevalence of smoking is major among men, and this can play a role in the higher severity of COVID-19 in men [118]. In cigarette smoking, there are polycyclic aromatic compounds, which can induce CYP enzymes (CYP1A1, CYP1A2, CYP2E1) and isoforms of uridine diphosphate glucuronosyltransferase and other drug-metabolizing enzymes [119,120,121]. Besides, tobacco smoke increases inflammation in a sex- and gender-dependent manner [31,122]. The above data indicate that the variables sex–gender and smoking habit should be included in the design and statistical analysis of clinical trials to reduce heterogeneity and to increase adherence to real life.

## 6. Can Male and Female COVID-19 Patients Have the Same Safety Profile as COVID-19 Free Patients?

All drugs may induce ADR. Spontaneous reports are essential for post-marketing surveillance, but they may cause several limitations including underreporting, variations in the quality of information, missing data, etc. [123]. The eventual sex–gender bias in reporting ADR has not been fully calculated [124,125]. Actually, it emerges that women have lower safety profiles [17,126,127,128,129]. Notably, most drugs have been excluded from the market because of their toxic effects, which have been described mainly in women ([20] and cited literature). Women seem to be admitted to hospitals for ADR more than men ([130], however, about this last point there are no univocal data [131,132]). Besides, women have a bigger immune response to vaccines than men [82,83], but they also have more common severe side effects [133,134,135]. Unfortunately, no sufficient attention has been paid to sexual dimorphism in vaccine clinical trials, including those for SARS-CoV-2 vaccines [136]. In addition, local and general ADR are being addressed yet are not segregated by gender [136,137,138]. However, a clinical trial of the adenovirus-vector vaccine candidate measured adverse effects outcomes and reported that females experienced ADR such as fever more commonly than males [138].

Utilizing VigiBase, Zekarias et al. [139] found that QT-prolongation has a rate of 31% and 19% in men and women with COVID-19, respectively. Whereas, in COVID-19-free patients, the QT-prolongation prevails in women [140]. Pro-inflammatory cytokines elevate the risk of QT-prolongation and fatal arrhythmias [141] and reduce the activity of CYP (Table 2). This is crucial because COVID-19 patients often have myocardial damage that might be a trigger for enhanced arrhythmic risk [142]. Both chloroquine and hydroxychloroquine are metabolized by CYP3A4 and, when they are used in combination with antiviral agents such as lopinavir/ritonavir, atazanavir, remdesivir, or other inhibitors of CYP3A4, the risk of QT-prolongation and drug-induced cardiac death could be enhanced [143]. 

Not all repurposed drugs prolong QT: tocilizumab and sarilumab, for example, can shorten it [144]. In addition, other sex differences in ADR with hydroxychloroquine and lopinavir/ritonavir are described. Hepatitis, diarrhea, nausea, vomiting, and other hepatic and kidney-related events are more reported in men, whereas, in women the most reported are diarrhea, nausea, vomiting, and upper abdominal pain [139]. Further, psychiatric ADR induced by hydroxychloroquine prevail in women in indications such as rheumatic diseases, systemic lupus erythematosus, or malaria [145], while, in COVID-19 patients, they prevail in men [146]. It is not known if the higher rate depends on a higher proportion of men treated by hydroxychloroquine, or by severe COVID-19 observed in men, which may promote, in turn, pharmacokinetic changes.

It is a still matter of discussion how risky is the use of non-steroidal anti-inflammatory drugs in COVID-19 [147].

These data suggest that our knowledge on drugs gained in COVID-19-free individuals is not readily transferable to patients with COVID-19, and this could produce a deterioration of the safety profile.

## 7. Conclusions

COVID-19 is a global health concern, which requires further investigations to identify the key players in sex and gender bias found in disease outcomes and, more importantly, in response to anti-viral treatment modalities including the drug safety profile.

Drug discovery and development is a long and complex challenge at varied levels such as the drug design, clinical setting, and the regulatory, intellectual property, and commercial levels. Therefore, because of the emergency determined by COVID-19, repurposing seems to be a good choice to accelerate the whole process. However, looking at the studies with repurposed drugs in COVID-19, it emerges that sex and gender have been neglected; although we are in front of a disease that presents significant sex and gender differences [148] and where the essential sexually dimorphic immune system plays a crucial role [53] decreasing, for example, drug-metabolizing enzymes and transporters activities leading to changes in the pharmacokinetics [104,105,106,111,117].

Therefore, it is necessary to promote more sex- and gender-sensitive research also in repurposing to have drugs that are equally safe and effective for women and men using sex and gender as a biological variable to optimize therapy in both men and women and to strengthen the use of gender medicine in daily clinical practice.

## Figures and Tables

**Table 1:** Pharmacokinetic of some protease inhibitors in the presence of ritonavir in non-COVID-19 patients: effect of sex.

Drug	Pharmacokinetic Parameters	Men vs. Women	References
Saquinavir	AUC _0–12h_ Cmin	25% higher in women 3-fold higher in women	[86]
	AUC _0–24h_, Cmin, Cmax	Higher in women	[87]
	AUC _0–24h_ Cmin, Cmax, CL	Higher in women NS	[88]
	AUC _0–12h_, Cmin, Cmax,	Higher in women with low significance	[89]
	AUC _0–24h_, Cmax,	Higher in women	[90]
Ritonavir	AUC _0–24h_, Cmax, Cmin, CL AUC, Cmax Median apparent oral CL	NS Higher in women Lower in women	[88] [91]
	AUC_0–12h_, Cmax	Higher in women	[87]
	AUC _0–24h_, Cmax,	Higher in women	[90]
Indinavir	CL, Cmin (after correction for deviation from 70 kg of body weight)	Lower in women Lower in women	[92]
Lopinavir	AUC _0–12h_, Cmin, Cmax	NS	[87]
Atazanavir	AUC _24h_, Cmax CL	NS Lower in women	[90] [93]
Darunavir	AUC _12h_	NS	[94]

AUC: area under the curve; CL: clearance; Cmin: the minimum blood plasma concentration reached by a drug before administration of a second dose; Cmax: the maximum (or peak) serum or plasma concentration that a drug achieves; NS: not significant.

**Table 2:** Examples of the effect of lipopolysaccharide and pro-inflammatory cytokines on human CYP enzymes CES1 and CES2, phase II enzymes, and drug transporters.

Targets	Inflammatory Triggers
CYP2C8, CYP3A4	LPS, TNF-α, IL-1β, IL-6
CYP1A2, CYP2B6, CYP2C9	IL-6, IFN-γ
CYP2B6	IFN-γ
CES1 and 2	IL-6
mRNA encoding CYP1A2, CYP2B6, CYP2E1, UGT2B7, SULT1A1, OAT2, CYP3A4, MRP2	IFN-a2B

CES: carboxylesterases; IFN: interferon; OAT2: organic anion transporter, SULT: sulfotransferase, UGT2B7: UDP-glucuronosyltransferase 2B7; multidrug MRP2: resistance-associated protein 2. Data from [104,105,106,107,117].

## Data Availability

Data sharing not applicable.

## References

[1] Gns H.S., Gr S., Murahari M., Krishnamurthy M. (2019). An update on drug repurposing: Re-written saga of the drug’s fate. Biomed. Pharm..

[2] Vane J.R. (1971). Inhibition of prostaglandin synthesis as a mechanism of action for aspirin-like drugs. Nat. New Biol..

[3] Chast F. (2009). Fabuleux Hasards—Histoire de la Découverte des Médicaments; Préface de Maurice Tubiana, C. Bohuon, C. Monneret. EDP Sciences, Les Ulis. Ann. Pharm. Françaises.

[4] Rothwell P.M., Fowkes F.G., Belch J.F., Ogawa H., Warlow C.P., Meade T.W. (2011). Effect of daily aspirin on long-term risk of death due to cancer: Analysis of individual patient data from randomised trials. Lancet.

[5] Socchi F., Bigorre M., Normandin M., Captier G., Bessis D., Mondain M., Blanchet C., Akkari M., Amedro P., Gavotto A. (2021). Hemangiol(R) in infantile haemangioma: A paediatric post-marketing surveillance drug study. Br. J. Clin. Pharm..

[6] Botting J. (2002). The History of Thalidomide. Drug News Perspect..

[7] Raje N., Anderson K. (1999). Thalidomide—A revival story. N. Engl. J. Med..

[8] Fintel B., Samaras A.T., Carias E. The Thalidomide Tragedy: Lessons for Drug Safety and Regulation. https://helix.northwestern.edu/article/thalidomide-tragedy-lessons-drug-safety-and-regulation.

[9] Ashburn T.T., Thor K.B. (2004). Drug repositioning: Identifying and developing new uses for existing drugs. Nat. Rev. Drug Discov..

[10] Jourdan J.P., Bureau R., Rochais C., Dallemagne P. (2020). Drug repositioning: A brief overview. J. Pharm. Pharm..

[11] Doan T.L., Pollastri M., Walters M.A., Georg G.I. (2011). The future of drug repositioning. Old drugs, new opportunities. Annual Reports in Medicinal Chemistry.

[12] Pushpakom S., Iorio F., Eyers P.A., Escott K.J., Hopper S., Wells A., Doig A., Guilliams T., Latimer J., McNamee C. (2019). Drug repurposing: Progress, challenges and recommendations. Nat. Rev. Drug Discov..

[13] Naylor S., Kauppi M., Schonfeld J.M. (2014). Therapeutic drug repurposing, repositioning and rescue: Part II: Business review. Drug Discov. World.

[14] Sarhan A.A., Ashour N.A., Al-Karmalawy A.A. (2021). The journey of antimalarial drugs against SARS-CoV-2: Review article. Inf. Med. Unlocked.

[15] Campesi I., Montella A., Franconi F. (2020). Letter to the Editor in response to the article ‘Candidate drugs against SARS-CoV-2 and COVID-19’. Pharmacol. Res..

[16] Cavalla D., Dudley J., Berliocchi L.E. (2016). Scientific commercial value of drug repurposing. Drug Repositioning—Approaches and Applications for Neurotherapeutics.

[17] Mauvais-Jarvis F., Berthold H.K., Campesi I., Carrero J.J., Dakal S., Franconi F., Gouni-Berthold I., Heiman M.L., Kautzky-Willer A., Klein S.L. (2021). Sex- and gender-based pharmacological response to drugs. Pharm. Rev..

[18] Zucker I., Prendergast B.J. (2020). Sex differences in pharmacokinetics predict adverse drug reactions in women. Biol. Sex. Differ..

[19] Rastegar-Mojarad M., Ye Z., Kolesar J.M., Hebbring S.J., Lin S.M. (2015). Opportunities for drug repositioning from phenome-wide association studies. Nat. Biotechnol..

[20] Franconi F., Campesi I., Colombo D., Antonini P. (2019). Sex-Gender variable: Methodological recommendations for increasing scientific value of clinical studies. Cells.

[21] Franconi F., Campesi I. (2017). Sex impact on biomarkers, pharmacokinetics and pharmacodynamics. Curr. Med. Chem..

[22] Madla C.M., Gavins F.K.H., Merchant H., Orlu M., Murdan S., Basit A.W. (2021). Let’s talk about sex: Differences in drug therapy in males and females. Adv. Drug Deliv Rev..

[23] (2018). European Institute for Gender Equality, Concepts and Definitions. https://eige.europa.eu/gender-mainstreaming/concepts-and-definitions.

[24] (2018). WHO, Gender, Equity and Human Rights. https://www.who.int/gender-equity-rights/understanding/gender-definition/en/.

[25] (2018). National Institute of Health, Sex & Gender. https://orwh.od.nih.gov/sex-gender.

[26] (2018). Australian Government, Australian Government Guidelines on the Recognition of Sex and Gender in Attorney General’s Department, Ed. https://www.ag.gov.au/Pages/default.aspx.

[27] Lopes-Ramos C.M., Chen C.Y., Kuijjer M.L., Paulson J.N., Sonawane A.R., Fagny M., Platig J., Glass K., Quackenbush J., DeMeo D.L. (2020). Sex differences in gene expression and regulatory networks across 29 human tissues. Cell Rep..

[28] Franconi F., Campesi I., Occhioni S., Tonolo G. (2012). Sex-gender differences in diabetes vascular complications and treatment. Endocr. Metab. Immune Disord. Drug Targets.

[29] Campesi I., Franconi F., Montella A., Dessole S., Capobianco G. (2021). Human umbilical cord: Information mine in sex-specific medicine. Life.

[30] LaVeist T.A. (2005). Disentangling race and socioeconomic status: A key to understanding health inequalities. J. Urban. Health.

[31] Campesi I., Montella A., Sotgiu G., Dore S., Carru C., Zinellu A., Palermo M., Franconi F. (2021). Combined oral contraceptives modify the effect of smoking on inflammatory cellular indexes and endothelial function in healthy subjects. Eur. J. Pharm..

[32] Campesi I., Milella L., Palermo M., Sotgiu G., Reggiardo G., Franconi F. (2020). Cigarette smoking affects the differences between male and female phenotypes. Am. J. Transl. Res..

[33] Madsen T., Bourjeily G., Hasnain M. (2017). Sex- and gender-based medicine: The need for precise terminology. Gend. Genome.

[34] Mark S. (2005). Sex- and gender-based medicine: Venus, Mars, and beyond. Gend. Med..

[35] Corella D., Coltell O., Portoles O., Sotos-Prieto M., Fernandez-Carrion R., Ramirez-Sabio J.B., Zanon-Moreno V., Mattei J., Sorli J.V., Ordovas J.M. (2019). A guide to applying the sex-gender perspective to nutritional genomics. Nutrients.

[36] Bairey Merz C.N., Regitz-Zagrosek V. (2014). The case for sex- and gender-specific medicine. JAMA Intern. Med..

[37] Marino M., Masella R., Bulzomi P., Campesi I., Malorni W., Franconi F. (2011). Nutrition and human health from a sex-gender perspective. Mol. Asp. Med..

[38] Ursin R.L., Shapiro J.R., Klein S.L. (2020). Sex-biased immune responses following SARS-CoV-2 infection. Trends Microbiol..

[39] Marquez E.J., Chung C.H., Marches R., Rossi R.J., Nehar-Belaid D., Eroglu A., Mellert D.J., Kuchel G.A., Banchereau J., Ucar D. (2020). Sexual-dimorphism in human immune system aging. Nat. Commun.

[40] (2021). Global Health 5050, The COVID-19 Sex-Disaggregated Data Tracker. https://globalhealth5050.org/the-sex-gender-and-covid-19-project/the-data-tracker/.

[41] Perez-Lopez F.R., Tajada M., Saviron-Cornudella R., Sanchez-Prieto M., Chedraui P., Teran E. (2020). Coronavirus disease 2019 and gender-related mortality in European countries: A meta-analysis. Maturitas.

[42] Gadi N., Wu S.C., Spihlman A.P., Moulton V.R. (2020). What’s sex got to do with COVID-19? Gender-based differences in the host immune response to coronaviruses. Front. Immunol..

[43] Gostin L.O., Hodge J.G., Wiley L.F. (2020). Presidential powers and response to COVID-19. JAMA.

[44] Lin K.J., Schneeweiss S., Tesfaye H., D’Andrea E., Liu J., Lii J., Murphy S.N., Gagne J.J. (2020). Pharmacotherapy for hospitalized patients with COVID-19: Treatment patterns by disease severity. Drugs.

[45] Heustess A.M., Allard M.A., Thompson D.K., Fasinu P.S. (2021). Clinical Management of COVID-19: A review ofpharmacological treatment options. Pharmaceuticals.

[46] Scavone C., Mascolo A., Rafaniello C., Sportiello L., Trama U., Zoccoli A., Bernardi F.F., Racagni G., Berrino L., Castaldo G. (2021). Therapeutic strategies to fight COVID-19: Which is the status artis?. Br. J. Pharmacol..

[47] Hoffmann M., Kleine-Weber H., Schroeder S., Kruger N., Herrler T., Erichsen S., Schiergens T.S., Herrler G., Wu N.H., Nitsche A. (2020). SARS-CoV-2 cell entry depends on ACE2 and TMPRSS2 and is blocked by a clinically proven protease inhibitor. Cell.

[48] Li Y., Zhang Z., Yang L., Lian X., Xie Y., Li S., Xin S., Cao P., Lu J. (2020). The MERS-CoV receptor DPP4 as a candidate binding target of the SARS-CoV-2 Spike. iScience.

[49] Iacobellis G. (2020). COVID-19 and diabetes: Can DPP4 inhibition play a role?. Diabetes Res. Clin. Pract..

[50] Sultana J., Crisafulli S., Gabbay F., Lynn E., Shakir S., Trifiro G. (2020). Challenges for drug repurposing in the COVID-19 pandemic era. Front. Pharm..

[51] (2020). NHI, COVID-19 Treatment Guidelines. https://www.covid19treatmentguidelines.nih.gov.

[52] He G., Massarella J., Ward P. (1999). Clinical pharmacokinetics of the prodrug oseltamivir and its active metabolite Ro 64-0802. Clin. Pharm..

[53] Manjili R.H., Zarei M., Habibi M., Manjili M.H. (2020). COVID-19 as an acute inflammatory disease. J. Immunol..

[54] Crisafulli S., Isgro V., La Corte L., Atzeni F., Trifiro G. (2020). Potential role of anti-interleukin (IL)-6 drugs in the treatment of COVID-19: Rationale, clinical evidence and risks. BioDrugs.

[55] Mauvais-Jarvis F. (2021). Do anti-androgens have potential as therapeutics for COVID-19?. Endocrinology.

[56] Montoya M.C., Krysan D.J. (2018). Repurposing estrogen receptor antagonists for the treatment of infectious disease. mBio.

[57] Ivanova M.M., Mazhawidza W., Dougherty S.M., Minna J.D., Klinge C.M. (2009). Activity and intracellular location of estrogen receptors alpha and beta in human bronchial epithelial cells. Mol. Cell Endocrinol..

[58] Getachew B., Tizabi Y. (2021). Vitamin D and COVID-19: Role of ACE2, age, gender, and ethnicity. J. Med. Virol..

[59] Cangiano B., Fatti L.M., Danesi L., Gazzano G., Croci M., Vitale G., Gilardini L., Bonadonna S., Chiodini I., Caparello C.F. (2020). Mortality in an Italian nursing home during COVID-19 pandemic: Correlation with gender, age, ADL, vitamin D supplementation, and limitations of the diagnostic tests. Aging.

[60] Correale J., Ysrraelit M.C., Gaitan M.I. (2010). Gender differences in 1,25 dihydroxyvitamin D3 immunomodulatory effects in multiple sclerosis patients and healthy subjects. J. Immunol..

[61] Sanghera D.K., Sapkota B.R., Aston C.E., Blackett P.R. (2017). Vitamin D status, gender differences, and cardiometabolic health disparities. Ann. Nutr. Metab..

[62] Campesi I., Romani A., Franconi F. (2019). The sex-gender effects in the road to tailored botanicals. Nutrients.

[63] Verdoia M., Schaffer A., Barbieri L., Di Giovine G., Marino P., Suryapranata H., De Luca G. (2015). Impact of gender difference on vitamin D status and its relationship with the extent of coronary artery disease. Nutr. Metab. Cardiovasc. Dis..

[64] Al-Horani H., Abu Dayyih W., Mallah E., Hamad M., Mima M., Awad R., Arafat T. (2016). Nationality, gender, age, and body mass index influences on vitamin D concentration among elderly patients and young Iraqi and Jordanian in Jordan. Biochem. Res. Int..

[65] Ya’qoub L., Elgendy I.Y., Pepine C.J. (2021). Sex and gender differences in COVID-19: More to be learned!. Heart J. Plus Cardiol. Res. Pract..

[66] Wehbe Z., Hammoud S.H., Yassine H.M., Fardoun M., El-Yazbi A.F., Eid A.H. (2021). Molecular and biological mechanisms underlying gender differences in COVID-19 severity and mortality. Front. Immunol..

[67] Oni T., Gideon H.P., Bangani N., Tsekela R., Seldon R., Wood K., Wilkinson K.A., Goliath R.T., Ottenhoff T.H., Wilkinson R.J. (2012). Smoking, BCG and employment and the risk of tuberculosis infection in HIV-infected persons in South Africa. PLoS ONE.

[68] Anker M. (2007). Addressing Sex and Gender in Epidemic-Prone Infectious Diseases.

[69] Peckham H., de Gruijter N.M., Raine C., Radziszewska A., Ciurtin C., Wedderburn L.R., Rosser E.C., Webb K., Deakin C.T. (2020). Male sex identified by global COVID-19 meta-analysis as a risk factor for death and ITU admission. Nat. Commun..

[70] Sudre C.H., Murray B., Varsavsky T., Graham M.S., Penfold R.S., Bowyer R.C., Pujol J.C., Klaser K., Antonelli M., Canas L.S. (2021). Attributes and predictors of Long-COVID. Nat. Med..

[71] Chamekh M., Casimir G. (2020). Understanding gender-bias in critically Ill patients with COVID-19. Front. Med. (Lausanne).

[72] Chen J., Bai H., Liu J., Chen G., Liao Q., Yang J., Wu P., Wei J., Ma D., Chen G. (2020). Distinct clinical characteristics and risk factors for mortality in female inpatients with Coronavirus Disease 2019 (COVID-19): A sex-stratified, large-scale cohort study in Wuhan, China. Clin. Infect. Dis..

[73] (2018). Gender Studies in Product Development: Historical Overview. https://www.fda.gov/science-research/womens-health-research/gender-studies-product-development-historical-overview.

[74] Curno M.J., Rossi S., Hodges-Mameletzis I., Johnston R., Price M.A., Heidari S. (2016). A systematic review of the inclusion (or exclusion) of women in HIV research: From clinical studies of antiretrovirals and vaccines to cure strategies. J. Acquir. Immune Defic. Syndr..

[75] Weinberger A.H., McKee S.A., Mazure C.M. (2010). Inclusion of women and gender-specific analyses in randomized clinical trials of treatments for depression. J. Women’s Health.

[76] Woitowich N.C., Beery A., Woodruff T. (2020). A 10-year follow-up study of sex inclusion in the biological sciences. eLife.

[77] Brady E., Nielsen M.W., Andersen J.P., Oertelt-Prigione S. (2021). Lack of consideration of sex and gender in clinical trials for COVID-19. Nat. Commun..

[78] Palmer-Ross A., Ovseiko P.V., Heidari S. (2021). Inadequate reporting of COVID-19 clinical studies: A renewed rationale for the Sex and Gender Equity in Research (SAGER) guidelines. BMJ Glob. Health.

[79] Clayton J.A., Collins F.S. (2014). Policy: NIH to balance sex in cell and animal studies. Nature.

[80] Ebina K., Hashimoto M., Yamamoto W., Hirano T., Hara R., Katayama M., Onishi A., Nagai K., Son Y., Amuro H. (2019). Drug tolerability and reasons for discontinuation of seven biologics in elderly patients with rheumatoid arthritis—The ANSWER cohort study. PLoS ONE.

[81] Burmester G.R., Rubbert-Roth A., Cantagrel A., Hall S., Leszczynski P., Feldman D., Rangaraj M.J., Roane G., Ludivico C., Lu P. (2014). A randomised, double-blind, parallel-group study of the safety and efficacy of subcutaneous tocilizumab versus intravenous tocilizumab in combination with traditional disease-modifying antirheumatic drugs in patients with moderate to severe rheumatoid arthritis (SUMMACTA study). Ann. Rheum. Dis..

[82] Harper A., Flanagan K.L. (2018). Effect of sex on vaccination outcomes: Important but frequently overlooked. Curr. Opin. Pharm..

[83] Fischinger S., Boudreau C.M., Butler A.L., Streeck H., Alter G. (2019). Sex differences in vaccine-induced humoral immunity. Semin. Immunopathol..

[84] Kees F., Bucher M., Schweda F., Gschaidmeier H., Faerber L., Seifert R. (2007). Neoimmun versus Neoral: A bioequivalence study in healthy volunteers and influence of a fat-rich meal on the bioavailability of Neoimmun. Naunyn Schmiedebergs Arch. Pharmacol..

[85] (2010). European Medicines Agency, Guideline on the Investigation of Bioequivalence. https://www.ema.europa.eu/en/investigation-bioequivalence.

[86] Fletcher C.V., Jiang H., Brundage R.C., Acosta E.P., Haubrich R., Katzenstein D., Gulick R.M. (2004). Sex-based differences in saquinavir pharmacology and virologic response in AIDS Clinical Trials Group Study 359. J. Infect. Dis..

[87] Ribera E., Lopez R.M., Diaz M., Pou L., Ruiz L., Falco V., Crespo M., Azuaje C., Ruiz I., Ocana I. (2004). Steady-state pharmacokinetics of a double-boosting regimen of saquinavir soft gel plus lopinavir plus minidose ritonavir in human immunodeficiency virus-infected adults. Antimicrob. Agents Chemother..

[88] Pai M.P., Schriever C.A., Diaz-Linares M., Novak R.M., Rodvold K.A. (2004). Sex-related differences in the pharmacokinetics of once-daily saquinavir soft-gelatin capsules boosted with low-dose ritonavir in patients infected with human immunodeficiency virus type 1. Pharmacotherapy.

[89] Dickinson L., Back D.J., Chandler B. The Impact of gender on saquinavir hard-gel/ritonavir (1000/100 mg bid) pharmacokinetics and PBMC transporter expression in HIV-1 infected individuals. Proceedings of the 6th International Workshop on Clinical Pharmacology of HIV Therapy.

[90] Becker S., Tse M., Sterman F. Pharmacokinetics of once-daily saquinavir hard-gel capsule with low-dose ritonavir or full-dose atazanavir in seronegative volunteers: ASPIRE I. Proceedings of the 12th Conference on Retroviruses and Opportunistic Infections.

[91] Umeh O.C., Currier J.S., Park J.G., Cramer Y., Hermes A.E., Fletcher C.V. (2011). Sex differences in lopinavir and ritonavir pharmacokinetics among HIV-infected women and men. J. Clin. Pharm..

[92] Csajka C., Marzolini C., Fattinger K., Decosterd L.A., Telenti A., Biollaz J., Buclin T. (2004). Population pharmacokinetics of indinavir in patients infected with human immunodeficiency virus. Antimicrob. Agents Chemother..

[93] Venuto C.S., Mollan K., Ma Q., Daar E.S., Sax P.E., Fischl M., Collier A.C., Smith K.Y., Tierney C., Morse G.D. (2014). Sex differences in atazanavir pharmacokinetics and associations with time to clinical events: AIDS Clinical Trials Group Study A5202. J Antimicrob. Chemother..

[94] Sekar V., Ryan R., Schaible D., Mazikewich A., Mrus J. Pharmacokinetic profile of darunavir (DRV) co-administered with low dose ritonavir in treatment experienced women and men: 4 week analysis in a substudy of the GRACE trial. Proceedings of the 9th International Workshop on Clinical Pharmacology of HIV Therapy (IWCPHIV).

[95] Morgan E.T. (2009). Impact of infectious and inflammatory disease on cytochrome P450-mediated drug metabolism and pharmacokinetics. Clin. Pharm. Ther..

[96] White N.J., Miller K.D., Churchill F.C., Berry C., Brown J., Williams S.B., Greenwood B.M. (1988). Chloroquine treatment of severe malaria in children. Pharmacokinetics, toxicity, and new dosage recommendations. N. Engl. J. Med..

[97] Roberts D.J., Hall R.I. (2013). Drug absorption, distribution, metabolism and excretion considerations in critically ill adults. Expert Opin. Drug Metab. Toxicol..

[98] Chang K.C., Bell T.D., Lauer B.A., Chai H. (1978). Altered theophylline pharmacokinetics during acute respiratory viral illness. Lancet.

[99] Yamaguchi A., Tateishi T., Okano Y., Matuda T., Akimoto Y., Miyoshi T., Kobayashi S., Koitabashi Y. (2000). Higher incidence of elevated body temperature or increased C-reactive protein level in asthmatic children showing transient reduction of theophylline metabolism. J. Clin. Pharm..

[100] Kraemer M.J., Furukawa C.T., Koup J.R., Shapiro G.G., Pierson W.E., Bierman C.W. (1982). Altered theophylline clearance during an influenza B outbreak. Pediatrics.

[101] Williams S.J., Baird-Lambert J.A., Farrell G.C. (1987). Inhibition of theophylline metabolism by interferon. Lancet.

[102] Lee E.B., Daskalakis N., Xu C., Paccaly A., Miller B., Fleischmann R., Bodrug I., Kivitz A. (2017). Disease-Drug Interaction of Sarilumab and Simvastatin in Patients with Rheumatoid Arthritis. Clin. Pharm..

[103] Schmitt C., Kuhn B., Zhang X., Kivitz A.J., Grange S. (2011). Disease-drug-drug interaction involving tocilizumab and simvastatin in patients with rheumatoid arthritis. Clin. Pharm. Ther..

[104] Chen C., Han Y.H., Yang Z., Rodrigues A.D. (2011). Effect of interferon-alpha2b on the expression of various drug-metabolizing enzymes and transporters in co-cultures of freshly prepared human primary hepatocytes. Xenobiotica.

[105] Aitken A.E., Morgan E.T. (2007). Gene-specific effects of inflammatory cytokines on cytochrome P450 2C, 2B6 and 3A4 mRNA levels in human hepatocytes. Drug Metab. Dispos..

[106] Dickmann L.J., Patel S.K., Rock D.A., Wienkers L.C., Slatter J.G. (2011). Effects of interleukin-6 (IL-6) and an anti-IL-6 monoclonal antibody on drug-metabolizing enzymes in human hepatocyte culture. Drug Metab. Dispos..

[107] Schoergenhofer C., Hobl E.L., Schellongowski P., Heinz G., Speidl W.S., Siller-Matula J.M., Schmid M., Sunder-Plassmann R., Stimpfl T., Hackl M. (2018). Clopidogrel in Critically Ill Patients. Clin. Pharmacol. Ther..

[108] Stanke-Labesque F., Gautier-Veyret E., Chhun S., Guilhaumou R. (2020). Inflammation is a major regulator of drug metabolizing enzymes and transporters: Consequences for the personalization of drug treatment. Pharm. Ther..

[109] Shi J., Wang X., Eyler R.F., Liang Y., Liu L., Mueller B.A., Zhu H.J. (2016). Association of oseltamivir activation with gender and carboxylesterase 1 genetic polymorphisms. Basic Clin. Pharm. Toxicol..

[110] https://medsafe.govt.nz/profs/Datasheet/a/Actemrainf.pdf.

[111] Schoergenhofer C., Jilma B., Stimpfl T., Karolyi M., Zoufaly A. (2020). Pharmacokinetics of lopinavir and ritonavir in patients hospitalized with Coronavirus Disease 2019 (COVID-19). Ann. Intern. Med..

[112] Gregoire M., Le Turnier P., Gaborit B.J., Veyrac G., Lecomte R., Boutoille D., Canet E., Imbert B.M., Bellouard R., Raffi F. (2020). Lopinavir pharmacokinetics in COVID-19 patients. J. Antimicrob. Chemother..

[113] Zanger U.M., Schwab M. (2013). Cytochrome P450 enzymes in drug metabolism: Regulation of gene expression, enzyme activities, and impact of genetic variation. Pharm. Ther..

[114] https://emedicine.medscape.com/article/166724-overview.

[115] Wiedermann C.J. (2021). Hypoalbuminemia as surrogate and culprit of infections. Int. J. Mol. Sci..

[116] Aziz M., Fatima R., Lee-Smith W., Assaly R. (2020). The association of low serum albumin level with severe COVID-19: A systematic review and meta-analysis. Crit. Care.

[117] Yang J., Shi D., Yang D., Song X., Yan B. (2007). Interleukin-6 alters the cellular responsiveness to clopidogrel, irinotecan, and oseltamivir by suppressing the expression of carboxylesterases HCE1 and HCE2. Mol. Pharmacol..

[118] Abate B.B., Kassie A.M., Kassaw M.W., Aragie T.G., Masresha S.A. (2020). Sex difference in coronavirus disease (COVID-19): A systematic review and meta-analysis. BMJ Open.

[119] Maideen N.M.P. (2019). Tobacco smoking and its drug interactions with comedications involving CYP and UGT enzymes and nicotine. World J. Pharm..

[120] Zevin S., Benowitz N.L. (1999). Drug interactions with tobacco smoking. Update Clin. Pharm..

[121] de Graan A.J., Loos W.J., Friberg L.E., Baker S.D., van der Bol J.M., van Doorn L., Wiemer E.A., van der Holt B., Verweij J., Mathijssen R.H. (2012). Influence of smoking on the pharmacokinetics and toxicity profiles of taxane therapy. Clin. Cancer Res..

[122] Ashare R.L., Wetherill R.R. (2018). The intersection of sex Differences, tobacco use, and inflammation: Implications for psychiatric disorders. Curr. Psychiatry Rep..

[123] Hazell L., Shakir S.A. (2006). Under-reporting of adverse drug reactions: A systematic review. Drug Saf..

[124] Turner R.M., Pirmohamed M. (2014). Cardiovascular pharmacogenomics: Expectations and practical benefits. Clin. Pharm. Ther..

[125] Carr D.F., Alfirevic A., Pirmohamed M. (2014). Pharmacogenomics: Current state-of-the-art. Genes.

[126] De Vries S.T., Denig P., Ekhart C., Burgers J.S., Kleefstra N., Mol P.G.M., van Puijenbroek E.P. (2019). Sex differences in adverse drug reactions reported to the National Pharmacovigilance Centre in the Netherlands: An explorative observational study. Br. J. Clin. Pharm..

[127] Watson S., Caster O., Rochon P.A., den Ruijter H. (2019). Reported adverse drug reactions in women and men: Aggregated evidence from globally collected individual case reports during half a century. EClinicalMedicine.

[128] Zopf Y., Rabe C., Neubert A., Gassmann K.G., Rascher W., Hahn E.G., Brune K., Dormann H. (2008). Women encounter ADRs more often than do men. Eur. J. Clin. Pharm..

[129] Rademaker M. (2001). Do women have more adverse drug reactions?. Am. J. Clin. Dermatol..

[130] Giardina C., Cutroneo P.M., Mocciaro E., Russo G.T., Mandraffino G., Basile G., Rapisarda F., Ferrara R., Spina E., Arcoraci V. (2018). Adverse drug reactions in hospitalized patients: Results of the FORWARD (Facilitation of Reporting in Hospital Ward) study. Front. Pharm..

[131] Holm L., Ekman E., Jorsater Blomgren K. (2017). Influence of age, sex and seriousness on reporting of adverse drug reactions in Sweden. Pharmacoepidemiol. Drug Saf..

[132] Montastruc J.L., Lafaurie M., de Canecaude C., Durrieu G., Sommet A., Montastruc F., Bagheri H. (2021). Fatal adverse drug reactions: A worldwide perspective in the World Health Organization pharmacovigilance database. Br. J. Clin. Pharmacol..

[133] Fink A.L., Klein S.L. (2018). The evolution of greater humoral immunity in females than males: Implications for vaccine efficacy. Curr. Opin. Physiol..

[134] Fink A.L., Klein S.L. (2015). Sex and gender impact immune responses to vaccines among the elderly. Physiology.

[135] Krammer F. (2020). SARS-CoV-2 vaccines in development. Nature.

[136] Jackson L.A., Anderson E.J., Rouphael N.G., Roberts P.C., Makhene M., Coler R.N., McCullough M.P., Chappell J.D., Denison M.R., Stevens L.J. (2020). An mRNA vaccine against SARS-CoV-2–Preliminary report. N. Engl. J. Med..

[137] Folegatti P.M., Ewer K.J., Aley P.K., Angus B., Becker S., Belij-Rammerstorfer S., Bellamy D., Bibi S., Bittaye M., Clutterbuck E.A. (2020). Safety and immunogenicity of the ChAdOx1 nCoV-19 vaccine against SARS-CoV-2: A preliminary report of a phase 1/2, single-blind, randomised controlled trial. Lancet.

[138] Bar-Zeev N., Moss W.J. (2020). Encouraging results from phase 1/2 COVID-19 vaccine trials. Lancet.

[139] Zekarias A., Watson S., Vidlin S.H., Grundmark B. (2020). Sex differences in reported adverse drug reactions to COVID-19 drugs in a global database of individual case safety reports. Drug Saf..

[140] Rabkin S.W. (2015). Impact of age and sex on QT prolongation in patients receiving psychotropics. Can. J. Psychiatry.

[141] Lazzerini P.E., Boutjdir M., Capecchi P.L. (2020). COVID-19, arrhythmic risk, and inflammation: Mind the gap!. Circulation.

[142] Driggin E., Madhavan M.V., Bikdeli B., Chuich T., Laracy J., Biondi-Zoccai G., Brown T.S., Der Nigoghossian C., Zidar D.A., Haythe J. (2020). Cardiovascular considerations for patients, health care workers, and health systems during the COVID-19 pandemic. J. Am. Coll. Cardiol..

[143] CredibleMeds^®^. https://crediblemeds.org/pdftemp/pdf/CombinedList.pdf.

[144] Lazzerini P.E., Acampa M., Capecchi P.L., Fineschi I., Selvi E., Moscadelli V., Zimbone S., Gentile D., Galeazzi M., Laghi-Pasini F. (2015). Antiarrhythmic potential of anticytokine therapy in rheumatoid arthritis: Tocilizumab reduces corrected QT interval by controlling systemic inflammation. Arthritis Care Res..

[145] Drew J. (1962). Concerning the side effects of antimalarial drugs used in the extended treatment of rheumatic disease. MJA.

[146] Garcia P., Revet A., Yrondi A., Rousseau V., Degboe Y., Montastruc F. (2020). Psychiatric disorders and hydroxychloroquine for Coronavirus Disease 2019 (COVID-19): A VigiBase study. Drug Saf..

[147] Capuano A., Scavone C., Racagni G., Scaglione F. (2020). NSAIDs in patients with viral infections, including COVID-19: Victims or perpetrators?. Pharmacol. Res..

[148] Pastor-Barriuso R., Perez-Gomez B., Hernan M.A., Perez-Olmeda M., Yotti R., Oteo J., Sanmartin J.L., Leon-Gomez I., Fernandez-Garcia A., Fernandez-Navarro P. (2020). Infection fatality risk for SARS-CoV-2: A nationwide seroepidemiological study in the non-institutionalized population of Spain. BMJ.

